# Programmable Fabrics of Enzyme-Responsive Amphiphiles: A Multiscale Platform for Hierarchical Mesophase Transformations

**DOI:** 10.1021/acs.biomac.4c01649

**Published:** 2025-05-07

**Authors:** Nicole Edelstein-Pardo, Shira Kutchinsky, Amit Sitt, Roey J. Amir

**Affiliations:** 1 School of Chemistry, Faculty of Exact Sciences, 26745 Tel-Aviv University, Tel-Aviv 6997801, Israel; 2 The Center for Physics and Chemistry of Living Systems, 26745 Tel-Aviv University, Tel Aviv 6997801, Israel; 3 Tel Aviv University Center for Nanoscience and Nanotechnology, 26745 Tel-Aviv University, Tel-Aviv 6997801, Israel; 4 The ADAMA Center for Novel Delivery Systems in Crop Protection, 26745 Tel-Aviv University, Tel Aviv 6997801, Israel

## Abstract

Systems capable of undergoing a controlled cascade of mesophase transitions across hierarchical scales represent a novel class of dynamic materials. Here, we describe an electrospun polymeric fabric composed of enzyme-responsive di- and triblock copolymers that undergoes a hierarchical cascade of four distinct mesophases. Initially, on immersion in water, the macroscale fabric dissolves, forming nanoscale micelles. Enzymatic degradation of the diblock components triggers a transition into a triblock-based hydrogel. Finally, the enzymatic degradation of the hydrogel into hydrophilic polymers leads to complete dissolution. By adjusting the di- and triblock ratios, we can finely tune the fabric’s dissolution rate. Moreover, the fibers can encapsulate hydrophobic agents, which are retained within the micelle and hydrogel phases, enabling their controlled release. This cascade of mesophase transitions, from a macroscopic solid to nanoscale assemblies, organized hydrogels, and eventual molecular dissolution, demonstrates sophisticated hierarchical control, unlocking new opportunities for biomedical applications of programmable materials.

## Graphical abstract

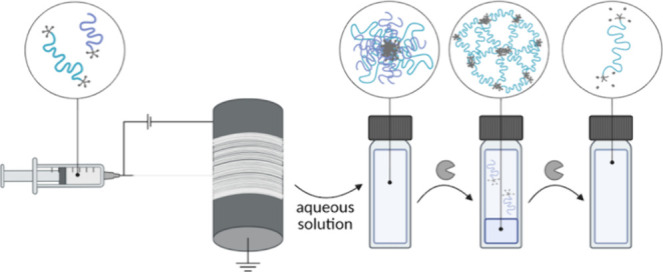

## Introduction

Stimuli-responsive polymers, which alter their shape, size, and chemical or physical properties in response to environmental changes, have long fascinated researchers because of their significant scientific value and promising applications.^1–5^ Polymers that respond to biochemical signals and cues gain particular focus due to the ability to incorporate them into biological systems and utilize them for numerous functions including sensing, tissue growth and engineering, signaling, and drug release.^6–11^ Among the variety of biochemical stimuli that have been utilized, enzymes have emerged as promising and highly potent triggers for activating stimuli-responsive polymers. Enzymes play a key role in almost every biological and metabolic process in living organisms, and each enzyme can be associated with specific roles in these processes. Many diseases are characterized by imbalances in the expression and activity of specific enzymes in the diseased tissue; thus, nonstandard overexpression of specific enzymes could be used for selective activation of advanced targeted drug delivery platforms.^12–14^

A leading approach for obtaining such delivery systems is the self-assembly of enzyme-responsive amphiphilic polymers into micelles, rods, and vesicles. There are different paths for molecular programming of the responsiveness of these enzyme-active systems. Although both bond formation and cleavage can be utilized, most enzyme-responsive polymers primarily rely on the latter as the mechanism for translating enzymatic activity into a material response. On cleavage by a specific enzyme, these self-assembled structures disassemble and release their content,^15–18^ making the degradation of these carriers highly beneficial for potential release applications.^13,19,20^ Examples of such a system include the disassembly of a polymeric system through the cleavage of an azo-based polymer by azoreductase in the presence of NADPH^21,22^ and the enzymatic cleavage of specific peptides by MMPs.^23–27^ For certain applications, enzymes play a secondary and indirect role. For instance, glucose oxidase is commonly used in glucose sensing, enabling the utilization of various types of pH-sensitive polymers for controlled insulin delivery.^28^ A key factor that affects the kinetics of the response and its efficiency is the architecture of the responsive polymer system. The relatively large size of enzymes restricts their penetration into the polymer bulk matrix, and as a result, the chemical interaction occurs mainly at the surface^29,30^ or by unimer-self-assembled structure equilibrium.^31^

When recruiting for solid and nonmobile polymeric systems, degradable mats and fibers fabricated by electrospinning hold great potential in the biomedical field.^32,33^ The notable characteristics of electrospun mats, including the fibers’ controllable diameters in the range of nanometers to micrometers, large specific surface area, and lightweight nature, have prompted their application for drug delivery, tissue engineering, and wound healing.^34–38^ Although the surface area-to-volume ratio of the fibers is larger than that of the polymeric bulk, the responsive sites inside the polymeric fibers are not directly accessible to the enzyme. Therefore, developing a stationary release system capable of controlled transition into mobile nanocarriers offers a combined benefit, integrating the strengths of both systems. In such a system, hydrophobic cargo such as therapeutic drugs can be easily encapsulated in the electrospun fabric. Once the drug loaded fabric transforms into a mobile system such as micelles that can maintain the therapeutic cargo, this mesophase transition can allow the further delivery of the hydrophobic cargo deeper into the tissue and finally its potential release in the target area in response to specific enzymatic stimuli.

Over the past decade, our group has utilized diblock amphiphiles (DBAs) based on hydrophilic polyethylene glycol (PEG) conjugated to a dendron with hydrophobic enzyme-responsive end-groups as a building block for the formation of enzymatically degradable assemblies. These amphiphiles, which are characterized by high molecular precision due to their dendritic blocks, self-assemble in an aqueous environment into spherical polymeric micelles that disassemble in response to the enzymatic hydrolysis of the hydrophobic end-groups.^39,40^ Extending this architecture of PEG-dendron DBAs to dendron-PEG-dendron triblock amphiphiles (TBAs), we demonstrated the use of these TBAs for the fabrication of electrospun polymeric fibers, which spontaneously broke into anisotropic particles due to the drying of the electrospun fibers. On placing these TBA-based microparticles in aqueous media, they transformed into hydrogel microparticles.^41^ More recently, by coassembling DBA and TBA, with an identical hydrophilic-to-lipophilic ratio in a 1:1 weight ratio, we obtained micelles that transformed between several distinct mesophases on exposure to an enzymatic stimulus, starting from nanoscale micelles, moving through bulk hydrogels, and finally to fully degraded polymers and complete dissolution.^42,43^

In this work, we report the development of a polymeric fabric that undergoes transitions between four different mesophases (Figure 1). The fabric is composed of electrospun fibers made of enzyme-responsive di- and triblock copolymers, which undergo a series of mesophase transitions from the microscale to the nanoscale. In the first stage, the solid fabric dissolves and forms nanosize micelles. Next, enzymatic degradation of the diblock components results in the destruction of the mixed micelles and the formation of a TBA-based hydrogel. Finally, a full dissolution of the system occurs through slow enzymatic degradation of the TBAs into soluble hydrophilic polymers. We examine the effect of the chemical composition of the formulation on the time frame of each transition stage and demonstrate the ability of the system to encapsulate and release hydrophobic cargo. This ability to program the transition from stationary to a mobile state and to control its dynamics opens the path for smart fabrics for controlled-release applications, such as drug-releasing dressings for wound healing applications.

**Figure 1:**
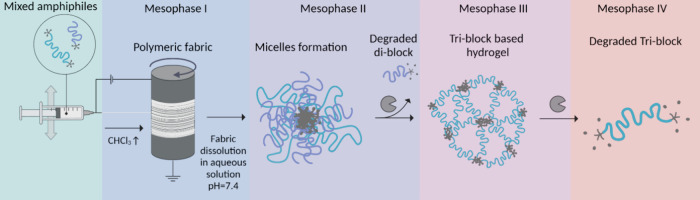
Schematic diagram of the proposed cascade mechanism showing the transition between four mesophases. Created in BioRender (Edelstein, N. (2025) https://BioRender.com/lb80y7e).

## Experimental Section

### Instrumentation

^1^H and ^13^C NMR spectra were recorded on a Bruker Avance III spectrometer (400 MHz for ^1^H NMR and 100 MHz for ^13^C NMR). Chemical shifts are reported in parts per million and referenced to the solvent. The molecular weights of the PEG-dendron diblock copolymers and dendron-PEG-dendron triblock copolymers were determined by comparison of the areas of the peaks corresponding to the PEG block (3.63 ppm) and the proton peaks of the dendrons.

#### Gel Permeation Chromatography (GPC)

All measurements were recorded on a Viscotek GPC Max instrument by Malvern using a refractive index detector. PEG standards (purchased from Sigma-Aldrich) were used for calibration.

#### Dynamic Light Scattering (DLS)

All measurements were recorded on a Corduan Technology VASCOγ particle size analyzer.

#### Scanning Electron Microscopy (SEM)

All images were taken using a Quanta 200FEG environmental SEM in high vacuum, WD ∼10 cm, 3–20 kV.

#### Spectrophotometer

Absorbance and fluorescence measurements were taken using a TECAN Infinite M200Pro using quartz cuvettes.

#### Fluorescence Spectrometer

Fluorescence measurements were recorded on an Agilent Technologies Cary Eclipse fluorescence spectrometer using quartz cuvettes.

### Materials

Poly­(ethylene glycol) (10 kDa and 5MDa), poly­(ethylene glycol) methyl ether (5 kDa), allyl bromide (99%), 2,2-dimethoxy-2-phenylacetophenone (DMPA, 99%), propargyl bromide 80% solution in toluene, 4-nitrophenol (99.5%), 4-dimethylaminopyridine (DMAP), *N*,*N*′-dicyclohexylcarbodiimide (DCC, 99%), 2-mercaptoethanol, nonanoic acid, Nile red, esterase from porcine liver (PLE), and Sephadex LH20 were purchased from Sigma-Aldrich. 3,5-Dihydroxybenzoic acid was purchased from Apollo Scientific Ltd. Anhydrous potassium carbonate was purchased from Alfa Aesar. Potassium hydroxide, cyclamine hydrochloride, and diisopropylethylamine (DIPEA) were purchased from Merck. Silica gel 60 Å 0.040–0.063 mm, sodium hydroxide, and all solvents were purchased from Bio-Lab and were used as received. Deuterated solvents for NMR were purchased from Cambridge Isotope Laboratories (CIL), Inc.

### Experimental Details

#### Fabric Electrospinning

The polymers’ jetting solutions were prepared by dissolving the amphiphilic custom-made copolymers (seven different ratios DBA:TBA 100:0, 75:25, 50:50, 45:55, 37.5:62.5, 25:75, 0:100) with a final concentration of 14% w/v and PEG-5MDa (0.8% w/v) in chloroform. The experimental setup for the electrospinning of the fabrics contained a power supply, a homemade rotary collector covered with nonstick aluminum foil, and a syringe pump placed on top of a moving X-stage. The polymer solution was dispensed via a syringe equipped with a 25-gauge needle. The solution was dispensed at a constant flow rate of 0.30–0.55 mL h^–1^. A driving voltage of 2.5–3.4 kV resulted in a stable jet, and the tip-to-collector distance was 15 cm. The collection was performed at room temperature in ambient conditions and a relative humidity of 70–75%. The syringe was repeatedly moved back and forth horizontally, resulting in alignment and uniform density of the deposited fibers.

#### Fabric Dissolution Rate Analysis

The dissolution analysis of the different fabrics was performed using HPLC. For each formulation, a piece of fabric weighing 6 mg was placed in a 4 mL glass vial, and 1 mL of 3 μM PLE in PBS solution was added to the vial. For each tested time point, a new sample was prepared. The samples were kept at 37 °C. For the HPLC measurements, the samples were filtered through a 0.22 μm nylon filter. The samples were injected into HPLC to monitor the amount of polymers in the solution at a certain time point. This data was collected by monitoring the area under the peak of the different components at 297 nm. Figure 2 reports the total concentration of DBA, which is the sum of the unhydrolyzed DBA and the hydrolyzed diblock at each time point. Figure S7 shows the concentration of the DBA and hydrolyzed diblock. The dissolution percentage of each copolymer was determined based on its initial content in the fabric, measured by HPLC after dissolving a fabric sample in acetonitrile at a concentration of 6 mg/mL. The experiments were performed in triplicate for all formulations.

**Figure 2:**
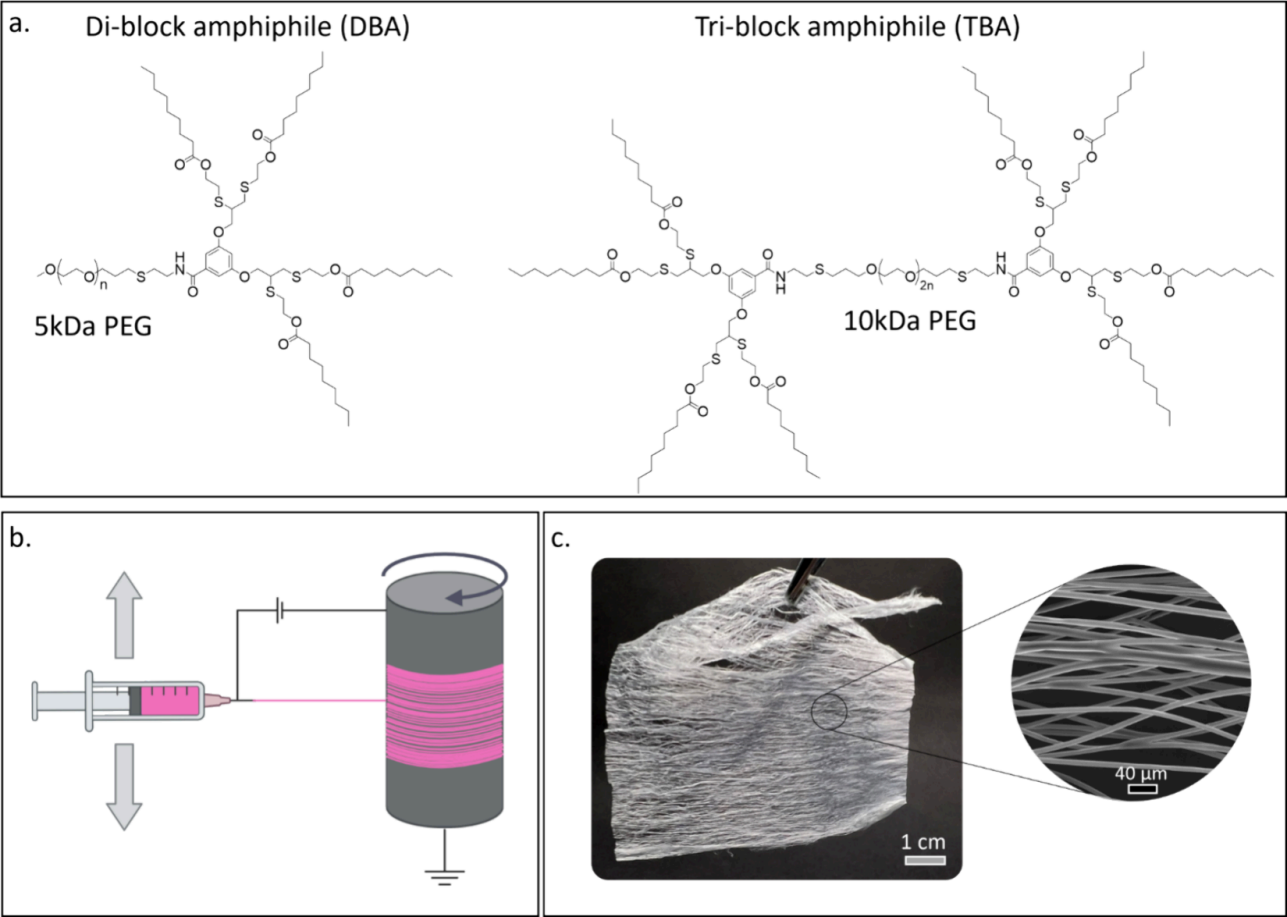
(a) Chemical structures of DBA and TBA synthesized in this work. (b) Schematic illustration of electrospinning over a rotating drum, used for the formation of the fabric. Created in BioRender (Edelstein, N. (2025) https://BioRender.com/lb80y7e). (c) Photo of the fabric and zoomed-in on the single fibers using scanning electron microscopy (SEM).

#### Micelle Formation Analysis by DLS

A 75 μL portion from the solution filtered by 0.22 μm nylon was tested in the DLS instrument to determine the arrangement in the solution at each time point. The experiments were performed in triplicate for all formulations.

#### Enzymatic Degradation of Micelle Analysis

The enzymatic degradation of micelles and the formation of hydrogel were monitored for the 50:50 DBA:TBA system. The solution with the dissolved fabric after 24 h was placed in a 1.5 mL HPLC vial, and degradation was followed at 37 °C by monitoring the area under the peak of TBA, DBA, and hydrolyzed diblock by HPLC at 297 nm. The experiment was performed in triplicate.

#### Hydrogel Degradation

To study the stability of the hydrogel formed from the enzymatic degradation experiment, we added BSA (50 mg/mL) and an excess of PLE (20 μM). A 21 mg portion of 50:50 DBA:TBA fabric was placed in a 4 mL vial, and 3.5 mL of 3 μM PLE in PBS solution was added. After 14 days at 37 °C, in which the two first transitions of fabric to micelles and micelles to hydrogel took place, the solution above the hydrogel was removed and replaced by the same volume of 20 μM PLE and 50 mg/mL BSA in PBS solution, kept at 37 °C, and manually stirred daily. The experiment was performed in triplicate.

#### Cargo Encapsulation and Release

The encapsulation of Nile red cargo was performed by the addition of the dye (5% mol/mol with respect to the total amount of copolymers) to an electrospinning solution of the 50:50 DBA:TBA formulation. The fabric was electrospun by the same procedure as with no Nile red added. Twenty-one milligrams of 50:50 DBA:TBA fabric was placed in a 4 mL quartz cuvette, and 3.5 mL of 3 μM PLE in PBS solution was added. The solution was kept at 37 °C, and absorbance measurements were taken according to the time points in Figure 5a. After 24 h, the cuvette was placed in a fluorimeter, and measurements were taken for 15 days. Then, for the hydrogel degradation, the solution above the hydrogel was removed and replaced with BSA and PLE solution as described before, and the sample was incubated at 37 °C and manually stirred daily. The experiments were performed in triplicate.

## Results and Discussion

To create responsive fabrics, we first synthesized PEG-based DBA and TBA (Figure 2a) composed of hydrophobic dendritic blocks containing enzymatically cleavable nonanoate end-groups. These aliphatic end-groups contained an ester bond, which can be broken by esterases. On hydrolysis, the nonanoic fatty acid chains are released, significantly reducing the hydrophobicity of the dendrons and exposing a degraded water-soluble hydrophilic PEG derivative (Figure S5.1).^40^

The formation of nonwoven fabrics from the synthesized DBAs and TBAs was performed via electrospinning. As schematically presented in Figure 2b, we used a rotary collector while moving the syringe needle back and forth to obtain sheets of nonwoven fabric (Figure 2c). In a typical fabrication, the copolymers were dissolved in chloroform at different DBA-to-TBA ratios, and a small amount of PEG-5MDa (0.8 w/v%) was added to the solution to increase the entanglement of the polymer chains and improve the spinning stability.^41^ Seven different fabric types were electrospun by using different formulations (Table 1). The obtained fabrics were cut into pieces, and each piece was placed in a glass vial, followed by the addition of a phosphate-buffered saline (PBS) solution with 3 μM porcine liver esterase (PLE), serving as a model enzyme, which can cleave the nonanoate end-groups. At different time intervals, the solution of the tested sample was filtered and analyzed using high-performance liquid chromatography (HPLC) to monitor the amount of polymers in the solution, while dynamic light scattering (DLS) was used to decipher their arrangement in the solution and in particular to monitor micelle formation.

**Table 1:** Electrospinning Formulation Parameters for the Different Samples

formulation ratio (DBA:TBA)	DBA (wt %)	TBA (wt %)	PEG-5MDa (wt %)
100:0	14	0	0.8
75:25	10.5	3.5	0.8
50:50	7	7	0.8
45:55	6.3	7.7	0.8
37.5:62.5	5.25	8.75	0.8
25:75	3.5	10.5	0.8
0:100	0	14	0.8

Figure 3 shows the dissolution profiles of fabrics made of fibers containing different DBA-to-TBA ratios. The dissolution percentage of each copolymer is calculated based on its initial amount in the fabric itself, measured by HPLC after fully dissolving a piece of fabric in acetonitrile at a concentration of 6 mg/mL. When the fabric is composed of only DBA, it dissolves almost immediately (within 5 min), resulting in the direct formation of micelles, which was indicated by DLS measurements (Figure 3a) and TEM micrographs (Figure S9.1). By adding TBA to the electrospinning formulation to a ratio of 75:25 DBA:TBA, the dissolution of the fibers becomes slower, and the fabric is fully dissolved after 2 h. Interestingly, when monitoring this system by HPLC, after 1 h, almost no polymers were observed in the solution, and the DLS measurement at this point shows objects in the size that corresponds to single polymeric chains and PLE. At the next measurement, at the time point of 2 h, we observed full dissolution and the formation of micelles (Figure 3b). The temporal dissolution profile of this fabric resembles a burst release profile of micelles from the fibers.

**Figure 3:**
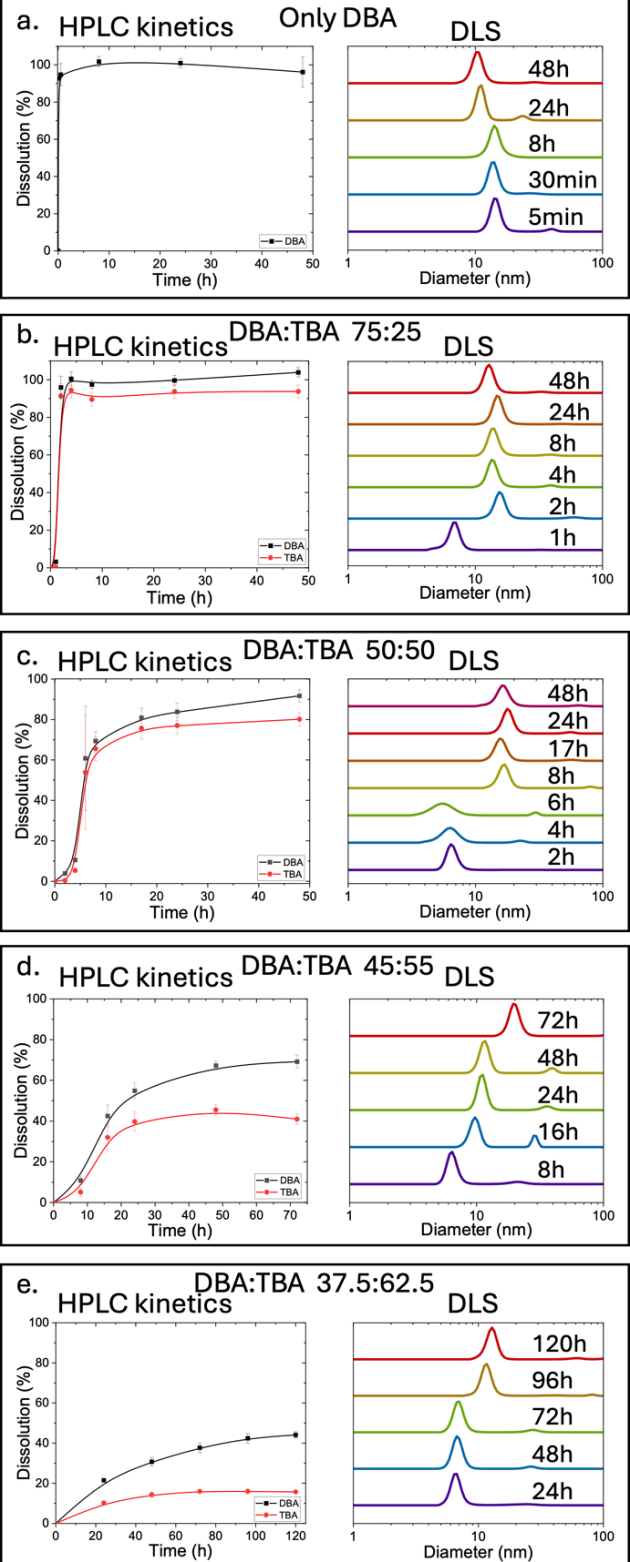
Dissolution profiles and DLS measurements of the different electrospun fabrics: (a) 100% DBA, (b) 75:25, (c) 50:50, (d) 45:55, and (e) 37.5:62.5 DBA:TBA ratios. For DBA, the reported concentration is the sum of the unhydrolyzed DBA and hydrolyzed diblock at each time point. The total concentration of fabric in solution is 6 mg/mL for all samples, and PLE concentration is 3 μM.

Fabrics made from a 50:50 DBA:TBA formulation showed even slower kinetics: micelles were formed only after 8 h, while full dissolution was observed only after 24 h (Figure 3c). The dissolution behavior of this 50:50 system exhibits a sigmoidal release profile with a short lag time, followed by faster release until reaching 70% dissolution and then a slower release rate to nearly 100% dissolution (SI Section S6). We believe that this is a result of a two-step process. In the initial stage of the dissolution process, the fibers undergo swelling, and only once the water content reaches a sufficient threshold, the fibers dissolve leading to the disassembly of the fabric and the formation of coassembled micelles. In the cases of fabrics made from formulations containing 25:75 DBA:TBA and only TBA, no polymer traces were obtained in the solution even when monitoring it for 2 weeks (Figure S10.1), indicating that for these formulations, the dissolution of the fabric is extremely slow and in practice negligible under the tested time frame.

To better understand the effect of the changes in the formulation on the dissolution rate and pattern, we focused on the region of a 50:50 ratio of DBA:TBA. We made an additional formulation with a DBA to TBA ratio of 45:55. It was striking to see that increasing the TBA portion by only 5% above the 50:50 ratio led to a significant reduction in the dissolution rate of the fiber as no full dissolution of the fabric was observed even after 3 days. The degree of dissolution for the individual DBA and TBA components for this 45:55 DBA:TBA ratio after 3 days was 70% and 40%, respectively. At this time point, we could observe a dual-phase system: swollen (hydrogel) fibers that contain 60% of the initial TBA and 30% of the initial DBA in the electrospinning formulation and micelles composed of 70% DBA and 40% TBA of the initial amounts (Figure 3d). The dissolution behavior of this 45:55 system also exhibits a sigmoidal release profile, but this system did not reach 100% dissolution (SI Section S6).

Furthermore, we tested a median ratio (37.5:62.5 DBA:TBA) between the 50:50 ratio, which showed dissolution within 24 h, and the 25:75 DBA:TBA ratio, which showed no dissolution (Figure S10.1). Similar to the 45:55 DBA:TBA ratio, no full dissolution was observed (Figure 3e). After 1 day, the amount of TBA that was found in the solution reached a maximum, while the amount of DBA showed a slow increase over time. Thus, in practice, the fabric made with this formulation exhibits a slow release of the DBA. Only once there was enough DBA in the solution, and its concentration rose above the critical micelle concentration (Figure S11.1), the formation of micelles was observed. Thus, the DBA release rate, controlled by its concentration in the fibers, acted as a “timer” for the formation of micelles.

These fabric dissolution experiments demonstrate the ability to control the dissolution rate and release profile by tuning the ratio of the two types of amphiphiles in the electrospinning formulations. The observed kinetic trends can be explained by considering that DBA serves as a dispersant for the TBA and hence affects its availability for forming mixed self-assembled micelles. Consequently, the ratio of DBA to TBA within the fibers serves as a controlling parameter for the dissolution kinetics. Fabrics made entirely of DBA dissolve rapidly, forming micelles. When TBA is added, the dissolution rate and micelle formation slow, depending on the DBA-to-TBA ratio. When the fabric contains more TBA than DBA, the DBAs cannot stabilize all the TBAs in the coassembled micellar form, and for this case, we observed two distinct systems. In the 45:55 DBA:TBA system, we obtained a two-phase system having micellar and hydrogel states simultaneously. When the amount of TBA is further increased, as in the 37.5:62.5 DBA:TBA formulation, the fabric also acts as a slow-release DBA platform. As shown in previous works and for the 100% TBA-based fabric, nonanoate-based TBAs do not dissolve in aqueous solution and instead form hydrogels.^41,42^

To examine the role of the PLE in this mesophase transition, we plotted the concentrations of DBA and its fully degraded product separately (Figure S7.1) and could clearly see that the dissolution rate is much faster than that of the enzymatic degradation. Additional control experiments with no addition of PLE were done for the 50:50 DBA:TBA formulation. These experiments showed the same trend of fabric dissolution, concluding that even in fibrous material, where the surface area-to-volume ratio is relatively high, the enzymatic degradation is rather slow (Figure S12.1).

While the presence of the enzyme was not found to affect the fibers’ dissolution, our previous work showed that it has a major effect at the micellar level. To examine this, we focused on the full degradation process of the 50:50 DBA:TBA-based fabric. Once we determined the dissolution pathways of the fabric, we set to study the subsequent enzymatically induced degradation of the DBA component and formation of a TBA-based hydrogel (Figure 4a). The photos in Figure 4b further validate that the first phase transition of the 50:50 DBA:TBA-based fabric to micelles takes place within the first 24 h. At this point, the solution is clear and contains micelles composed of DBA and TBA and a small amount of hydrolyzed diblock, which was formed by PLE (Figure 4d,e). From this time point, the concentration of the hydrolyzed diblock continues to increase while the amount of intact DBA decreases, and the TBA concentration remains nearly constant for 6 days.

**Figure 4:**
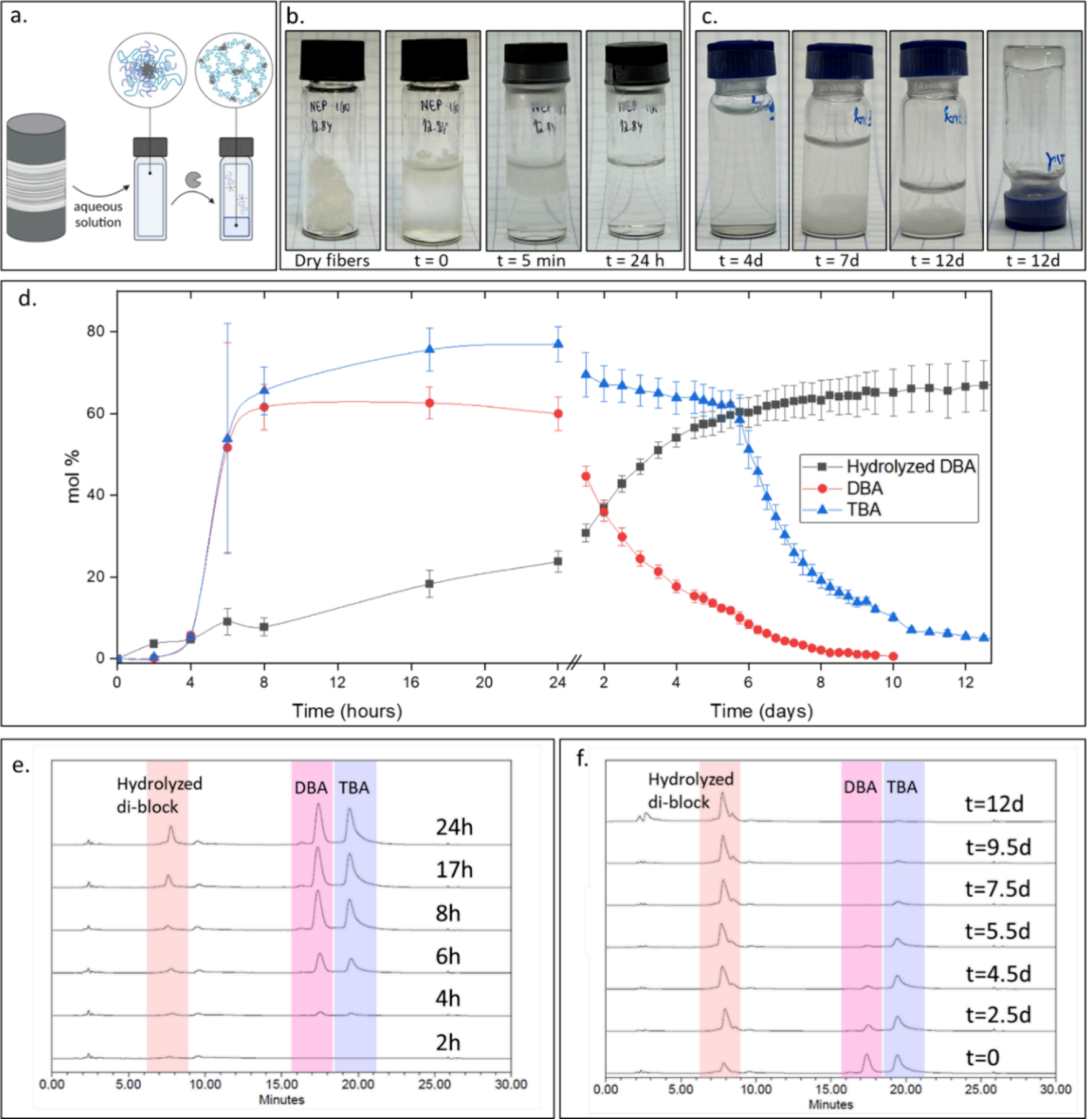
(a) Schematic illustration of the first and second transitions, from fabric into micelles and then to a hydrogel. Created in BioRender (Edelstein, N. (2025) https://BioRender.com/lb80y7e). (b) Photos of the fabric when soaked in the aqueous solution at a concentration of 6 mg/mL and after the full dissolution has taken over 24 h. (c) Photos of the formation of hydrogel over 12 days in 3 μM PLE in PBS solution. (d) Kinetics of the dissolution of the fabric at the first 24 h and the enzymatic degradation of the micelles due to cleavage of the hydrophobic end-groups. (e) Overlay of HPLC chromatograms of the first transition from fabric to micelles. (f) Overlay of HPLC chromatograms of the second transition from micelles to hydrogel.

After 6 days, the second mesophase transformation occurred, and the TBA concentration in the solution sharply decreased due to the formation of TBA-based hydrogel, which precipitated at the bottom of the vial (Figure 4c). The concentration of TBA continued to decrease in the following days, but no hydrolyzed triblock was observed (Figure 4d,f), indicating that the decrease in its concentration is due to hydrogel formation and not because of its enzymatic hydrolysis. At the same time, DBA’s concentration continued to decrease due to its enzymatic degradation, and simultaneously, the area of the peak for the hydrolyzed diblock kept increasing. In addition to the decrease in the DBA concentration due to enzymatic hydrolysis, a small amount of DBA accumulates in the TBA-based hydrogel (Figure S13.1).

The last transition in the multiphase cascade is from the hydrogel to soluble polymers (Figure 5a). Despite having the same nonanoate end-groups for the TBA, the hydrolysis of the ester bonds by PLE in the hydrogel could be expected to be even slower than the rate for the DBA degradation. This difference emerges from the slower kinetics of unimer-assembly exchange for TBA due to its molecular architecture^44^ and higher molecular weight in comparison with the DBA.^45,46^ Hence, to speed up the last mesophase transition, we increased the PLE concentration to 20 μM and added bovine serum albumin (BSA) to a concentration of 50 mg/mL. BSA, which acts as a transport protein in the blood and is known to interact nonspecifically with hydrophobic molecules and polymeric chains, was added to the solution to increase the concentration of the TBA in its unimer form and make it more accessible to the degrading enzyme.^47^ Figure 5b shows the transformation of the hydrogel to soluble degraded hydrophilic polymers, as indicated by the formation of a clear solution over a period of more than one month.

**Figure 5:**
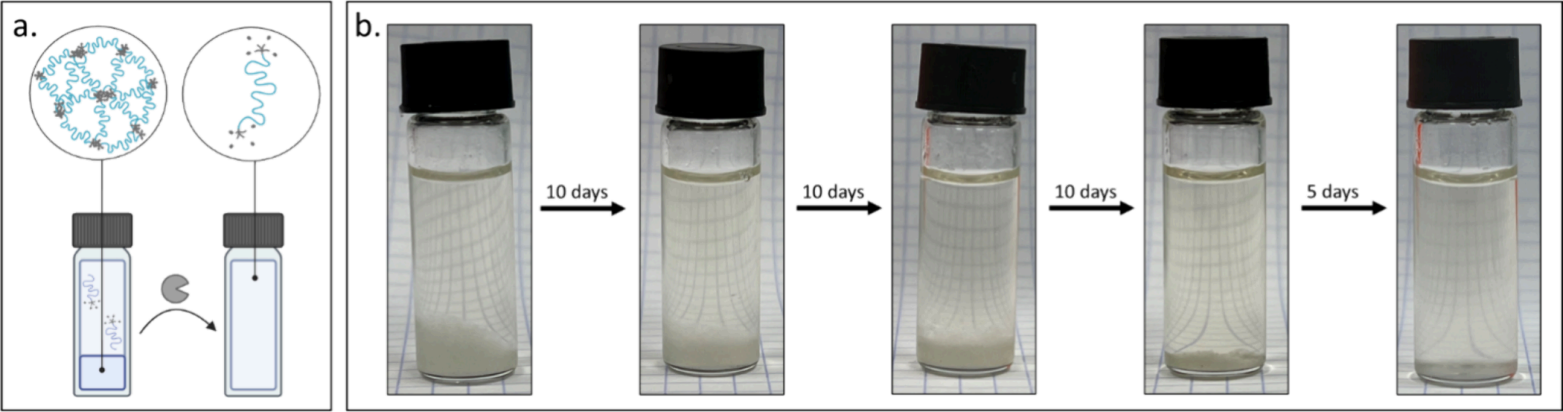
(a) Schematic illustration of the third transition, hydrogel dissolution. Created in BioRender (Edelstein, N. (2025) https://BioRender.com/lb80y7e). (b) Photos of the vial over time showing the final transition from hydrogel to soluble polymers and clear solution in the presence of PLE (20 μM) and BSA (50 mg/mL) in PBS.

The obtained results demonstrate the ability to prepare fabrics that can undergo a programmable cascade of mesophase transitions from solid fabric to micelles, then to hydrogel, and finally to fully dissolved polymers. Such fabrics can be extremely beneficial for biomedical applications, such as external and internal drug-eluting wound dressings, because the ability to program the timing of the transitions can be utilized to tune the release rate of encapsulated therapeutic cargo from the different mesophases. To demonstrate the ability of these fabrics to serve for the encapsulation and release of hydrophobic cargo, we incorporated Nile red as a model cargo into the electrospinning solution, indicated by the coloration of the fabric in a strong pink color (Figure 6a).

**Figure 6:**
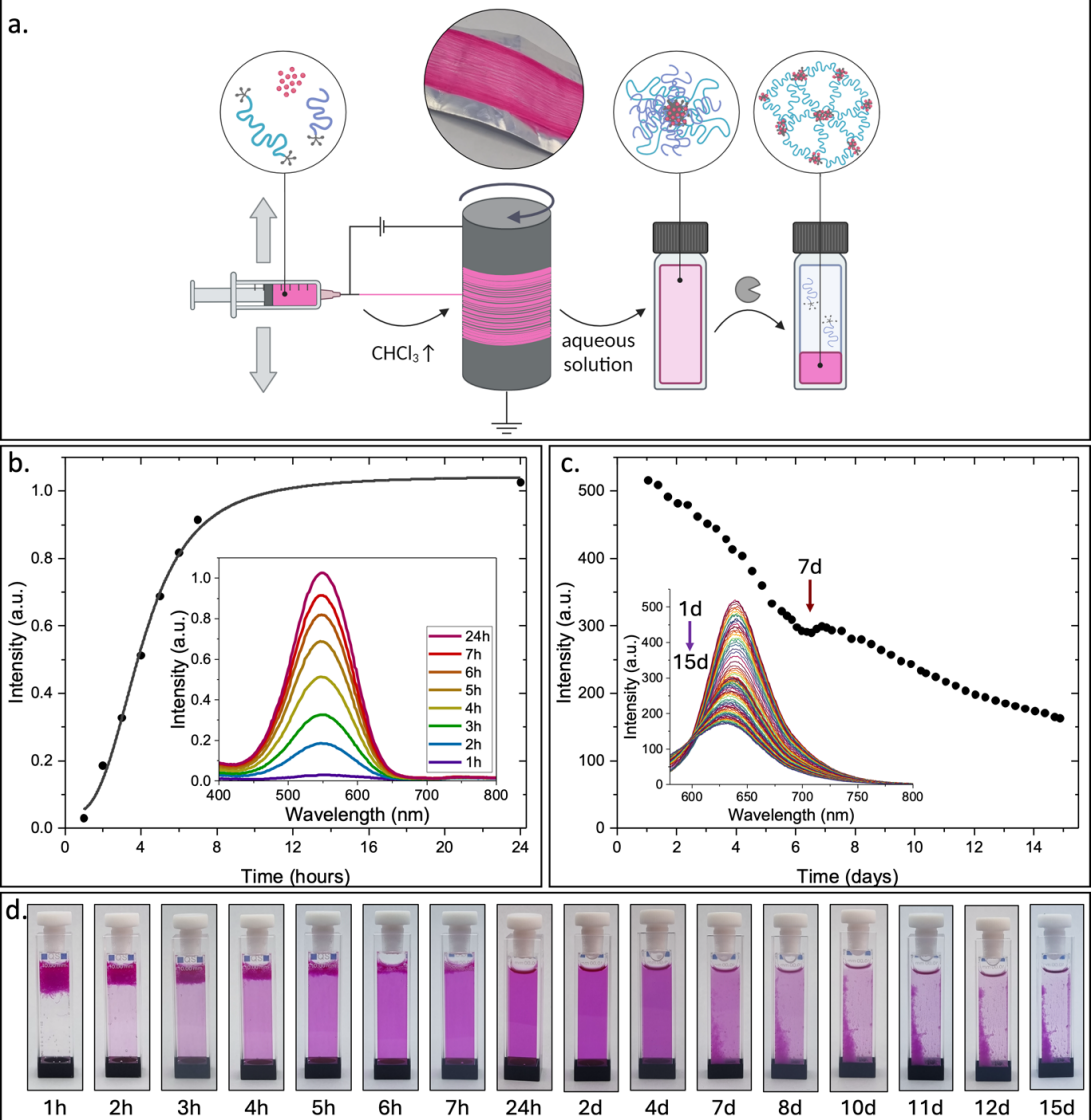
(a) Schematic illustration of Nile red encapsulation process by electrospinning followed by the dissolution of the fabric in aqueous solution (6 mg/mL fabric in 3 μM PLE in PBS) and the disassembly of the micelles to form a hydrogel. Created in BioRender (Edelstein, N. (2025) https://BioRender.com/lb80y7e). (b) Absorbance spectra of Nile red in the solution at the first transition. (c) Fluorescence spectra of Nile red at the second transition. (d) Photos of the cuvette during the process.

Using the Nile red-loaded 50:50 DBA:TBA-based fabric, we followed the absorbance of Nile red in the solution during the fabric to micelle transition (Figure 6b). The full dissolution of the fibers into micelles loaded with Nile red, as indicated by the increase in absorbance in the solution, was observed after 24 h. For the next step in the cascade of mesophase transformations, we followed the decrease in the fluorescent emission of Nile red during the transition of micelles to hydrogel (Figure 6c), and in this case, we observed the appearance of a hydrogel with a strong pink color after 7 days (Figure 6d). These results go in line with the HPLC results for the dye-free fabric in Figure 4. This encapsulation experiment demonstrates the feasibility of loading the polymeric fabric with hydrophobic cargo by simply adding it to the formulation before electrospinning. The low fluorescence of the solution and strong coloring of the hydrogel indicate its ability to maintain the encapsulated dye during the mesophase transition from micelles to the hydrogel.

Next, the solution above the Nile red-loaded hydrogel was changed to a solution of BSA (50 mg/mL) and PLE (20 μM) to monitor the hydrogel degradation. Figure 7 shows the route for the final dissolution of the system. After the solution exchange, the fluorescence intensity of Nile red gradually decreased due to sedimentation of the suspended hydrogel particles. However, we also noticed significant discoloration of the hydrogel, which is accompanied by phase separation of the dye and its aggregation. After 10 days, an increase in the Nile red concentration in the solution was observed both visually and by fluorescence measurements. This was due to the presence of hydrolyzed triblock and its end-groups in the solution that increases the solubility of Nile red, which has low solubility in a solution containing only BSA and PLE (Figure S14.1). In the following days, a further increase in fluorescence was observed as well as the disappearance of the hydrogel at the bottom of the cuvette. These results indicated the occurrence of the last mesophase transition on enzymatic degradation by PLE and the consequent dissolution of the TBA hydrogel into soluble hydrophilic polymers.

**Figure 7:**
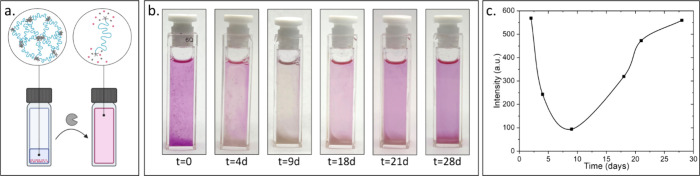
(a) Schematic illustration of the hydrogel encapsulating Nile red dissolution. Created in BioRender (Edelstein, N. (2025) https://BioRender.com/lb80y7e). (b) Photos of the cuvette over time. (c) Nile red fluorescence intensity over time. PLE (20 μM) and BSA (50 mg/mL) in PBS.

These preliminary encapsulation and release results demonstrate the potential applicability of such fabrics, which can be programmed to undergo a cascade of mesophase transitions for biomedical applications. We envision the use of such fabrics as dissolvable bandages that can allow the penetration of the micellar nanocarriers into the underlying tissue, where they can transform into hydrogel-based drug depots, enabling prolonged sustained release of their drug cargo before being fully degraded and allowing their clearance from the body after completing their therapeutic role.

## Conclusions

To conclude, we demonstrated the development of an electrospun enzyme-responsive fabric that can undergo a cascade of multimesophase transitions in response to a single enzymatic stimulus. At the first transition, from polymeric microfibers to micelles, the rate of transition and dissolution profile can be controlled by changing the ratio between DBA and TBA in the formulation of the electrospinning solution. We showed that we can achieve different dissolution profiles, from immediate release where full dissolution was observed after 2 h to sustained release that occurred over 24 h, and displayed a system that can act as a fabric that slow-releases micelles. For the second transition, from micelles to hydrogel, enzymatic activation causes the relatively slow degradation of the micelles by the selective hydrolysis of the DBA, resulting in the formation of the TBA-based hydrogel. The third transition, from hydrogel to soluble polymers, occurs for a longer period and results in the final phase of the system. To demonstrate the potential of the system in the field of controlled release, we encapsulated the hydrophobic cargo in the electrospun fabric and monitored its release through phase transitions.

Overall, this programmable system opens new frontiers for the design and fabrication of responsive polymeric materials that operate across different scales, from macroscopic fabrics to microscopic nanocarriers and hydrogels. The hierarchical nature of this cascade of mesophase transitions allows for the development of multifunctional fabrics that can transform into nanocarriers or biodegradable hydrogels for prolonged drug release. These advanced fabrics could be specifically used in medical applications such as bioresponsive bandages and stitches, offering targeted, controlled, and sustained therapeutic effects.

## Supplementary Material



## Notes

Detailed experimental information, description of the synthesis, characterization data, control experiments, and release profile analysis (PDF)
