# Supplementary material for: Programmable Fabrics of Enzyme-Responsive Amphiphiles: A Multiscale Platform for Hierarchical Mesophase Transformations
Source: Biomacromolecules. 2025 May 7;26(6):3281–90. doi: 10.1021/acs.biomac.4c01649 (PMC12152838; doi:10.1021/acs.biomac.4c01649)
Supplement: Supplementary file 1 [file bm4c01649_si_001.pdf]

Supporting information

Programmable Fabrics of Enzyme-Responsive Amphiphiles: A Multiscale Platform for Hierarchical Mesophase Transformations

Nicole Edelstein-Pardo[1,2,3], Shira Kutchinsky[1], Amit Sitt[1,2,3,4], Roey J. Amir[1,2,3,4]

[1]School of Chemistry, Faculty of Exact Sciences, Tel-Aviv University, Tel-Aviv 6997801, Israel

[2]The Center for Physics and Chemistry of Living Systems, Tel-Aviv University, Tel Aviv 6997801, Israel

[3]Tel Aviv University Center for Nanoscience and Nanotechnology, Tel-Aviv University, Tel-Aviv 6997801, Israel

[4]The ADAMA Center for Novel Delivery Systems in Crop Protection, Tel-Aviv University, Tel Aviv 6997801, Israel

Table of contents

S1. General information…..……………………………………………..………………....…S1

S2. Experimental details...……………………………………………………….……...…S2

S3. Detailed synthesis………………………………………………………….……...…S4

S4. Gel permeation chromatography (GPC) data……………………………..…………….....S8

S5. The amphiphiles degradation products……………….……………………..……..……….S9

S6. The sigmoidal release profile analysis……………………………………..…………….S11

S7. DBA and hydrolyzed di-block dissolution profiles…………………………………...…….S12

S8. DLS of PLE in PBS solution……………………………...……………………………S13

S9. Micelles analysis by Transmission Electron Microscopy (TEM)……………………………S13

S10. Undissolvable fabrics ……………..……………………………………………..………....S14

S11. Critical Micelle Concentration (CMC) Measurements………………………..…………S14

S12. Fabrics dissolution control……………..…………………………………..………….……...S15

S13. Hydrogel characterization …………..……...…………………………...……..…..…….....S16

S14. Nile red solubility test ……………..……………………………...….………………..…...S17

S1. General information

1. Instrumentation

^{1}H and ^{13}C-NMR: spectra were recorded on a Bruker Avance III spectrometer (400MHz for ^{1}H-NMR and 100MHz for ^{13}C-NMR). Chemical shifts are reported in ppm and referenced to the solvent. The molecular weights of the PEG-dendron di-block copolymers and dendron-PEG-dendron tri-block copolymers were determined by comparison of the areas of the peaks corresponding to the PEG block (3.63 ppm) and the protons peaks of the dendrons.

Gel permeation chromatography (GPC): All measurements were recorded on Viscotek GPC max by Malvern using a refractive index detector. PEG standards (purchased from Sigma-Aldrich) were used for calibration.

Dynamic light scattering (DLS): All measurements were recorded on a Corduan Technology VASCOγ particle size analyzer.

Scanning electron microscopy (SEM): All images were taken using a Quanta 200FEG environmental SEM in high vacuum, WD ~10cm, 3-20kV.

Spectrophotometer: Absorbance and fluorescence measurements were taken using TECAN Infinite M200Pro using quartz cuvettes.

Fluorescence spectrometer: Fluorescence measurements were recorded on an Agilent Technologies Cary Eclipse Fluorescence Spectrometer using quartz cuvettes.

2. Materials

Poly(Ethylene Glycol) (10kDa and 5MDa), Poly(ethylene glycol) methyl ether (5kDa), Allyl bromide (99%), 2,2-dimethoxy-2- phenylacetophenone (DMPA, 99%), Propargyl bromide 80% solution in toluene, 4-nitrophenol (99.5%), 4-dimethylamino pyridine (DMAP), N,N'-dicyclohexylcarbodiimide (DCC, 99%), 2-mercaptoethanol, Nonanoic acid, Nile Red, Esterase from porcine liver (PLE), and Sephadex® LH20 were purchased from Sigma-Aldrich. 3,5 dihydroxy benzoic acid was purchased from Apollo Scientific Ltd. Anhydrous potassium carbonate was purchased from Alfa Aesar. Potassium hydroxide, Cystamine hydrochloride, and Diisopropylethylamine (DIPEA) were purchased from Merck. Silica Gel 60Å 0.040-0.063mm, Sodium hydroxide, and all solvents were purchased from Bio-Lab and were used as received. Deuterated solvents for NMR were purchased from Cambridge Isotope Laboratories (CIL), Inc.

S2. Experimental details

1. Fabric Electrospinning

The polymers' jetting solutions were prepared by dissolving the amphiphilic custom-made copolymers (seven different ratios DBA:TBA 100:0, 75:25, 50:50, 45:55, 37.5:62.5, 25:75, 0:100) with a final concentration of 14% w/v, and PEG-5MDa (0.8% w/v) in chloroform. The experimental setup for the electrospinning of the fabrics contained a power supply, a homemade rotary collector covered with nonstick aluminum foil, and a syringe pump placed on top of a moving X-stage. The polymer solution was dispensed via a syringe equipped with a 25-gauge needle. The solution was dispensed at a constant flow rate of 0.30-0.55 mL h^{-1}. A driving voltage of 2.5-3.4 kV resulted in a stable jet, and the tip-to-collector distance was 15 cm. The collection was performed at room temperature in ambient conditions and a relative humidity of 70-75%. The syringe was repeatedly moved back and forth horizontally, resulting in alignment and uniform density of the deposited fibers.

2. Fabrics dissolution rate analysis

The dissolution analysis of the different fabrics was performed using HPLC. For each formulation, a piece of fabric weighing 6 mg was placed in a 4mL glass vial and 1mL of 3µM PLE in PBS solution was added to the vial. For each tested time point, a new sample was prepared. The samples were kept at 37°C. For the HPLC measurements, the samples were filtered through a 0.22µm nylon filter. The samples were injected into HPLC to monitor the amount of polymers in the solution at a certain time point. This data was collected by monitoring the area under the peak of the different components at 297nm. In Figure 2 the total concentration for DBA reported is the sum of the DBA and hydrolyzed di-block at each time point, the non-summed data is shown in Figure S7. The dissolution percentage of each copolymer was determined based on its initial content in the fabric, measured by HPLC after dissolving a fabric sample in acetonitrile at a concentration of 6 mg/mL. The experiments were performed in triplicates for all formulations.

3. Micelles formation analysis by DLS

75 µL from the solution filtered by 0.22 µm nylon was tested in the DLS instrument to determine the arrangement in the solution at each time point. The experiments were performed in triplicates for all formulations.

4. Enzymatic degradation of micelles analysis

For the 50:50 DBA:TBA system, we monitored the enzymatic degradation of micelles and the formation of hydrogel. The solution with the dissolved fabric after 24 hours was placed in an HPLC 1.5mL vial and degradation was followed at 37°C by monitoring the area under the peak of TBA, DBA, and hydrolyzed di-block by HPLC at 297 nm. The experiment was performed in triplicates.

5. Hydrogel degradation

To study the stability of hydrogel formed from the enzymatic degradation experiment we added BSA (50mg/mL) and an excess of PLE (20µM). 21 mg of 50:50 DBA:TBA fabric was placed in a 4mL vial and 3.5mL of 3µM PLE in PBS solution was added. After 14 days at 37°C, in which the two first transitions of fabric to micelles and micelles to hydrogel took place, the solution above the hydrogel was removed and replaced by the same volume of 20µM PLE and 50mg/mL BSA in PBS solution, kept at 37°C and manually stirred daily. The experiment was performed in triplicates.

6. Cargo encapsulation and release

The encapsulation of Nile red cargo was performed by the addition of the dye (5% mol/mol with respect to the total amount of copolymers) to the electrospinning solution of the 50:50 DBA:TBA formulation. The fabric was electrospun by the same procedure as with no Nile red added. 21 mg of 50:50 DBA:TBA fabric was placed in a 4 mL quartz cuvette and 3.5 mL of 3µM PLE in PBS solution was added. The solution was kept at 37°C and absorbance measurements were taken according to the time points in Figure 5a. After 24 hours, the cuvette was placed in a fluorimeter, and measurements were taken for 15 days. Then, for the hydrogel degradation, the solution above the hydrogel was removed and replaced with BSA and PLE solution as described before and the sample was incubated at 37°C and manually stirred daily. The experiments were performed in triplicates.

S3. **Detailed synthesis**

Synthesis of di-block amphiphiles (DBA)

mPEG5k-[dend-(nonanoate)$_4$] (DBA) was synthesized as previously reported and the spectroscopic characterization correlated well with this report.[1]

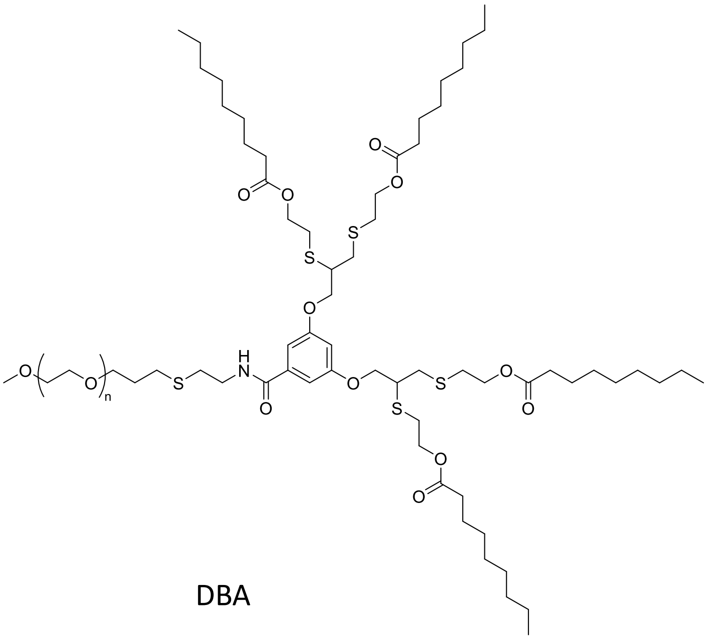

DBA

Figure S3.1: The chemical structure of DBA.

Synthesis of tri-block amphiphiles (TBA)

Bis-PEG10k-[dend-(yne)$_2$] was synthesized as previously reported and the spectroscopic characterization correlated well with this report.[2]

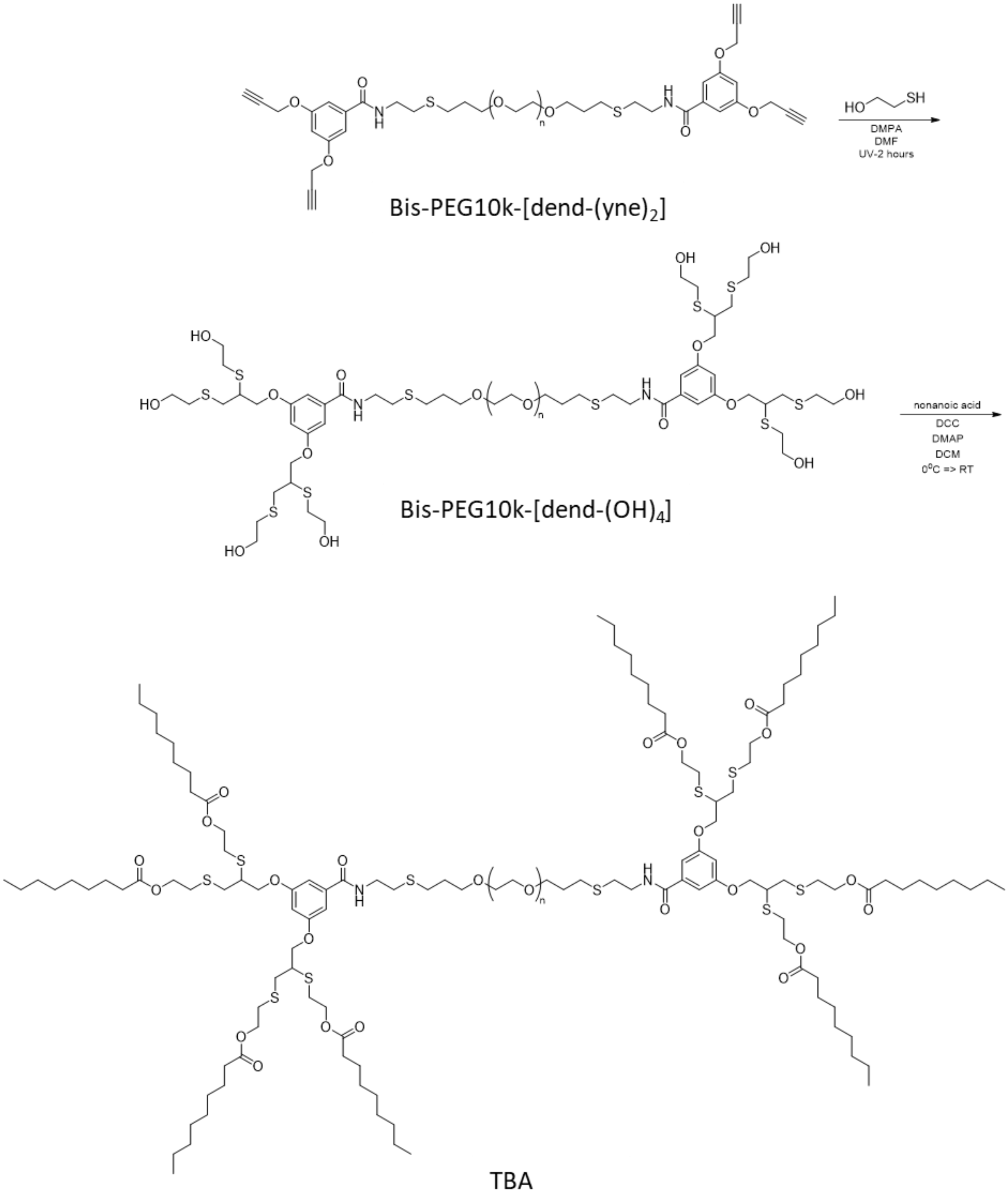

Figure S3.2: General preparation of TBA.

Bis-PEG10k-[dend-(OH)$_4$] synthesis:

Bis-PEG10k-[dend-(yne)2] (1g, 1eq.), 2-mercaptoethanol (160eq.), and DMPA (1.6eq.) were dissolved in DMF (5mL per 1g PEG). The solution was purged with nitrogen for 30 minutes and then placed under UV light at 365nm for 2 hours. The reaction was loaded as-is onto a MeOH-based LH20 column (Sephadex). Fractions that contained the product (identified with UV or I_2 coloring) were unified and the MeOH was evaporated to dryness. The products were obtained as white solids (quantitative yield). ^{1}H-NMR ($CDCl_3$): δ 7.11 (t, J = 5.8 Hz, 2H, -N***H***-CO-), 7.03 (d, J=2.3 Hz, 4H, Ar-***H***), 6.65 (t, J=2.3 Hz, 2H, Ar-***H***), 4.36 – 4.09 (m, 8H, Ar-O-C***H***$_2$), 3.89-3.41 (m, 1189H, PEG backbone + -C***H***$_2$-OH), 3.30 (qui, J = 6.4 Hz, 4H, -O-CH$_2$-C***H***-S-), 3.11 – 2.72 (m, 32H, -CH$_2$-S-CH$_2$-C***H***$_2$-NH- + -CH$_2$-S-C***H***$_2$-CH$_2$-NH- + -CH-C***H***$_2$-S-CH$_2$-CH$_2$-OH + -S-C***H***$_2$-CH$_2$-OH), 2.67 (t, J = 7.2 Hz, 4H, -C***H***$_2$-S-CH$_2$-CH$_2$-NH-), 1.88 (qui, J = 6.8 Hz, 4H, -O-CH$_2$-C***H***$_2$-CH$_2$-S-).^{13}C-NMR ($CDCl_3$): 167.5, 159.7, 135.9, 106.9, 105.0, 77.9, 71.2, 70.6, 61.7, 61.4, 45.3, 40.9, 35.2, 34.1, 32.1, 29.6, 28.3, 23.2; GPC (DMF + 25 mM NH_4Ac): expected M_n=11.2kDa, experimental M_n = 11.0 kDa, $Đ_M$=1.11;

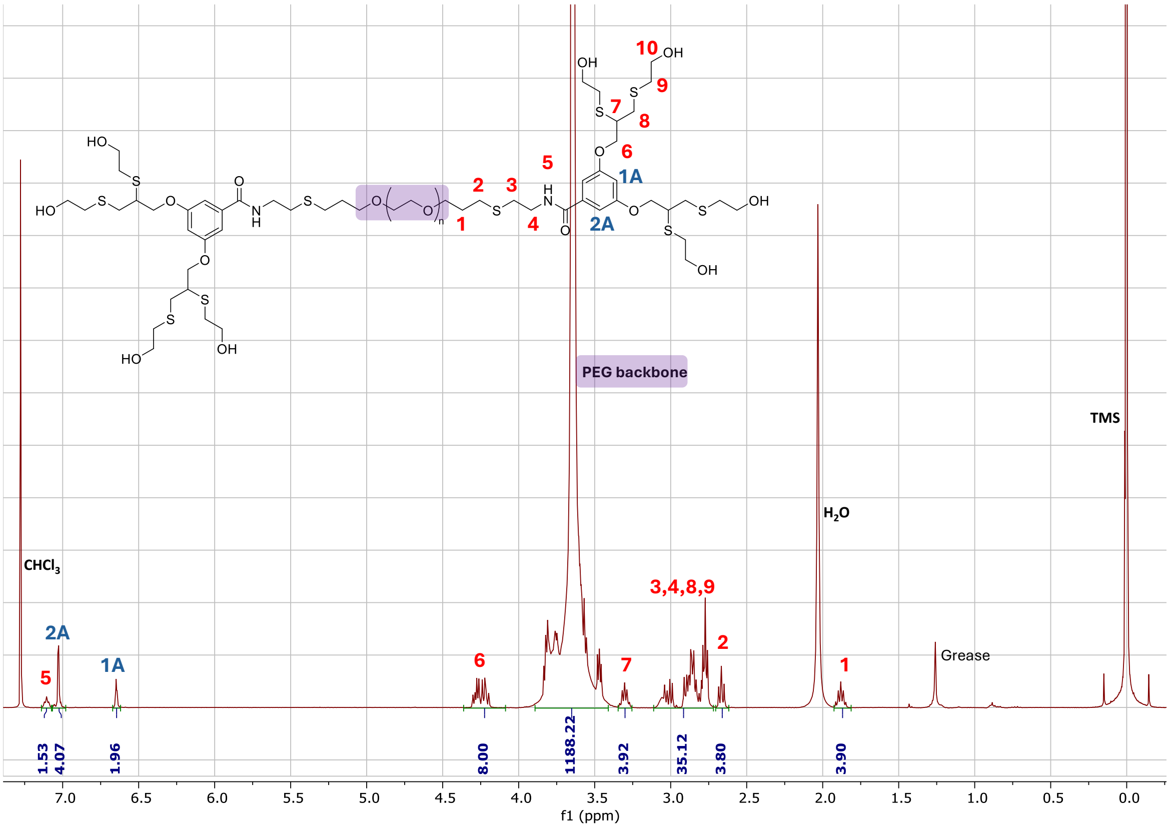

Figure S3.3: ^{1}H-NMR spectra of Bis-PEG10k-[dend-(OH)4] in $CDCl_3$.

Bis-PEG10k-[dend-(nonanoate)$_4$] (TBA) synthesis:

Bis-PEG10k-[dend-(OH)4] (1g, 1eq.) was dissolved in DCM (10mL) and nonanoic acid (30eq.) was added. The solution was cooled to 0°C followed by the addition of DCC (30eq.) and DMAP (1eq.). The reaction was stirred overnight and allowed to reach ambient temperature. Urea by-product was filtered off and washed with DCM. The solvent was evaporated to dryness and then the crude mixture was re-dissolved in a minimum amount of DMF and loaded onto a MeOH-based LH20 column (Sephadex). Fractions that contained the product (identified with UV or I_2 coloring) were unified and the MeOH was evaporated to dryness. The products were obtained as white solids (86% yield).

^{1}H-NMR ($CDCl_3$): δ 7.11 – 6.81 (m, 6H, -CH$_2$-N***H***-CO- +Ar-***H***), 6.59 (t, J = 2.3 Hz, 2H, Ar-***H***), 4.35 – 4.10 (m, 24H, Ar-O-C***H***$_2$- + -S-CH$_2$-C***H***$_2$-O-CO-), 3.84-3.44 (m, 1000H, PEG backbone), 3.19 (qui, J = 6.0 Hz, 4H, -O-CH$_2$-C***H***-S-), 3.06 – 2.51 (m, 36H, -C***H***$_2$-S-CH$_2$-CH$_2$-NH- + -CH$_2$-S-C***H***$_2$-CH$_2$-NH- + -CH$_2$-S-CH$_2$-C***H***$_2$-NH- + -CH-C***H***$_2$-S-CH$_2$-CH$_2$-O-CO- + -S-C***H***$_2$-CH$_2$-O-CO-), 2.29 (t, J=7.6 Hz, 16H, -O-CO-C***H***$_2$-(CH$_2$)$_6$-CH$_3$), 1.87 (qui, J = 6.4 Hz, 4H, -O-CH$_2$-C***H***$_2$-CH$_2$-S-), 1.67-1.53 (m, 16H, -O-CO-CH$_2$-C***H***$_2$-), 1.34-1.17 (m, 80H, -O-CO-CH$_2$-CH$_2$-(C***H***$_2$)$_5$-CH$_3$) , 0.85 (t, J=6.7 Hz, 24H, -CH$_2$-C***H***$_3$); ^{13}C-NMR ($CDCl_3$): δ 172.8, 166.9, 159.6, 136.9, 106.3, 104.7, 77.9, 70.6, ,64.3 64.1, 45.5, 39.3, 35.4, 34.9, 34.8, 31.9, 31.6, 29.7, 29.4, 29.3, 29.2, 28.7, 28.1, 25.8, 22.6, 14.11; GPC (DMF + 25 mM NH_4Ac): expected M_n=12.4kDa, experimental M_n = 11.9 kDa, $Đ_M$=1.14;

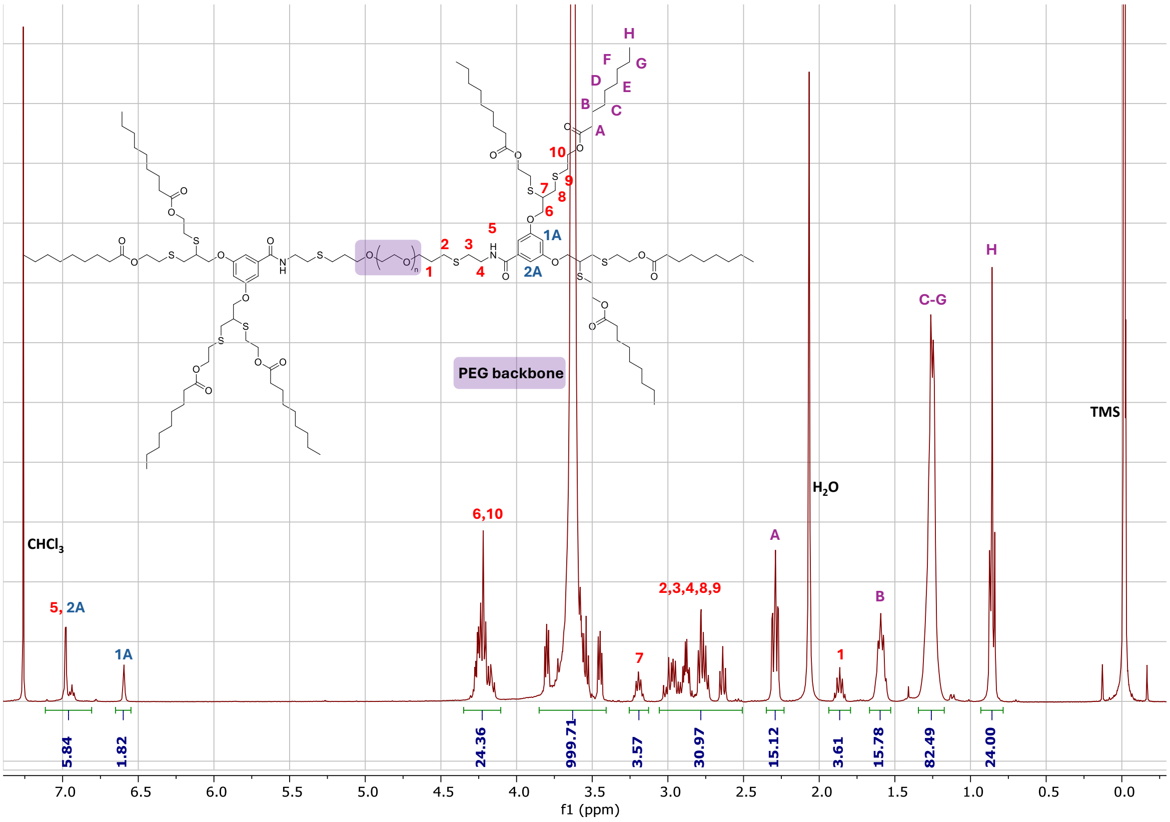

Figure S3.4: ^{1}H-NMR spectra of TBA in $CDCl_3$.

S4. <u>Characterization of amphiphilic tri-block copolymers by Gel Permeation Chromatography (GPC)</u>

Instrument method:

Instrument: Malvern Viscotek GPCmax

Columns: 2xPSS GRAM 1000Å

Columns temperature: 50°C

Flow rate: 0.5ml/min

Injection time: 60min

Injection volume: 50 µL from a 10 mg/ml sample

Diluent + mobile phase: DMF + 25mM NH_4Ac

Needle wash: DMF

Detector: Viscotek VE3580 RI detector

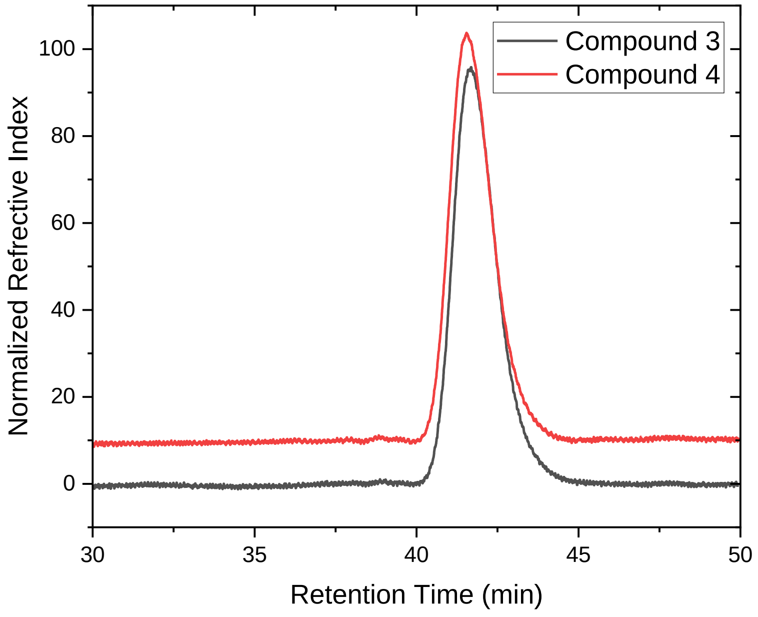

Figure S4.1: GPC overlay of the synthesized compounds.

S5. <u>The amphiphile degradation products</u>

Upon enzymatic hydrolysis of the amphiphiles, the nonanoic acid chains are released, which reveals a degraded, water-soluble hydrophilic PEG derivative with alcohol groups in the periphery (Figure S5.1 and S5.2).

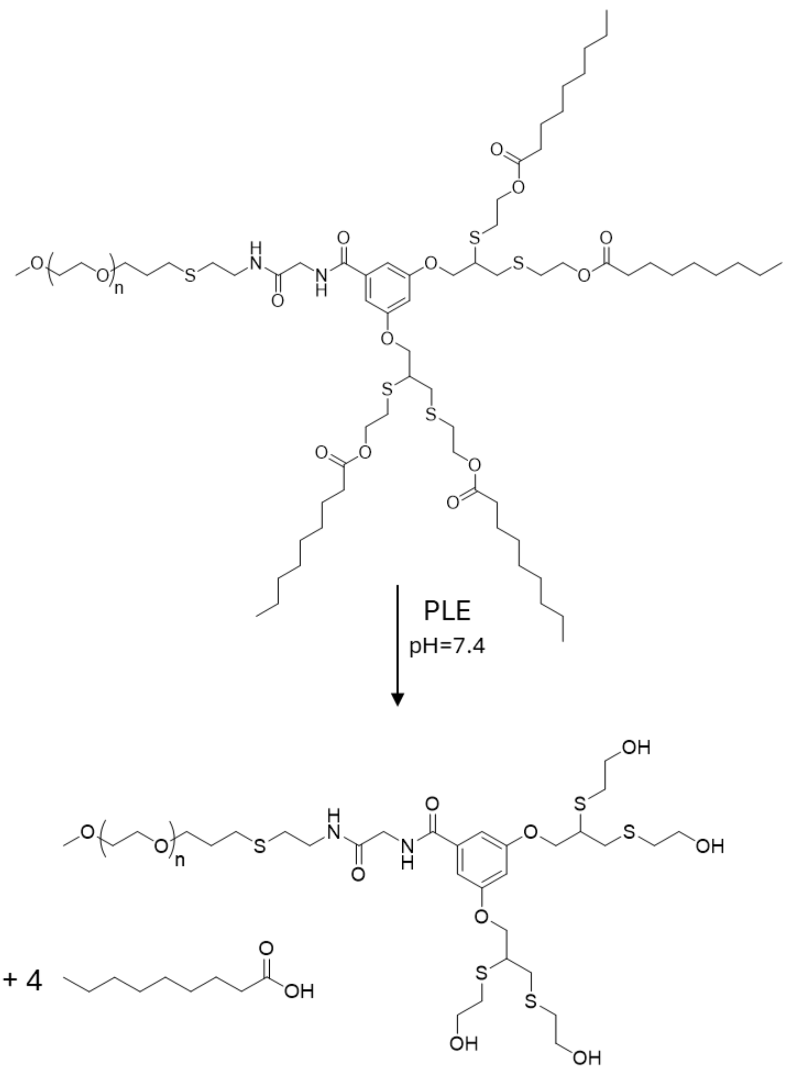

Figure S5.1: Enzymatic hydrolysis of DBA by PLE.

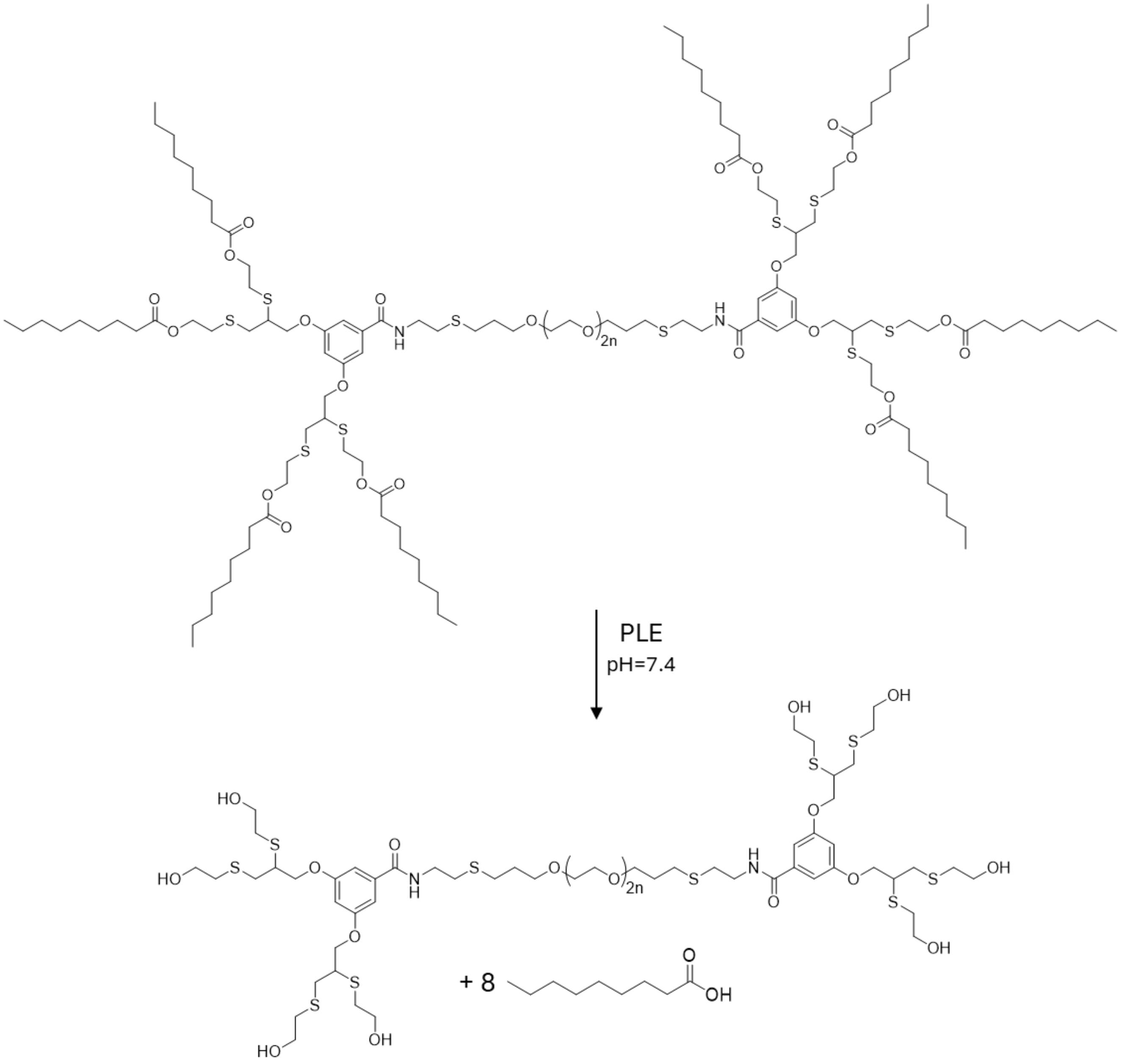

Figure S5.2: Enzymatic hydrolysis of TBA by PLE.

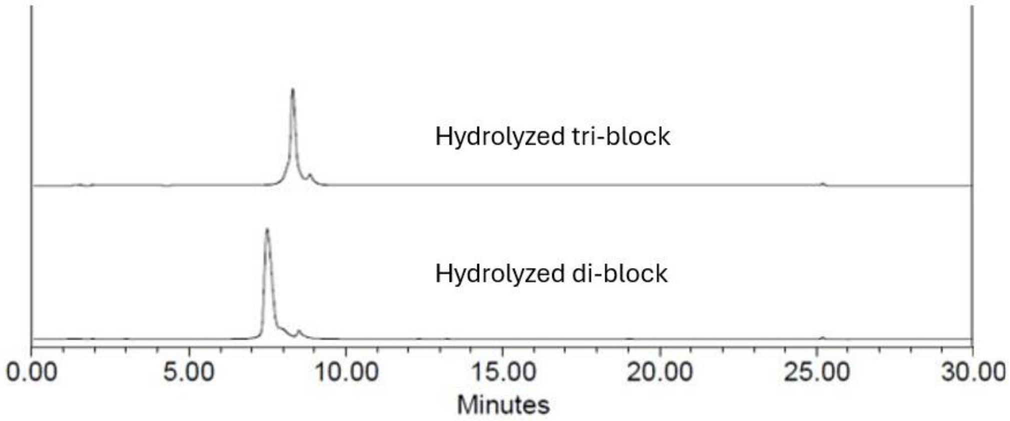

Figure S5.3: HPLC graphs of the hydrolyzed di-block and hydrolyzed tri-block.

S6. <u>The sigmoidal release profile analysis</u>

For the fabrics made of the 50:50 DBA:TBA and 45:55 DBA:TBA formulations the dissolution profiles resembled a sigmoidal shape curve. For analyzing this behavior, the experimental data was fitted to the four-parameter logistic model (dose-response model, Figure S6.1) which is commonly used to describe molecular release mechanisms.[3–6]. The analysis and fitting were done using OriginPro 2024 and the fitting parameters are presented in Table S6.1.

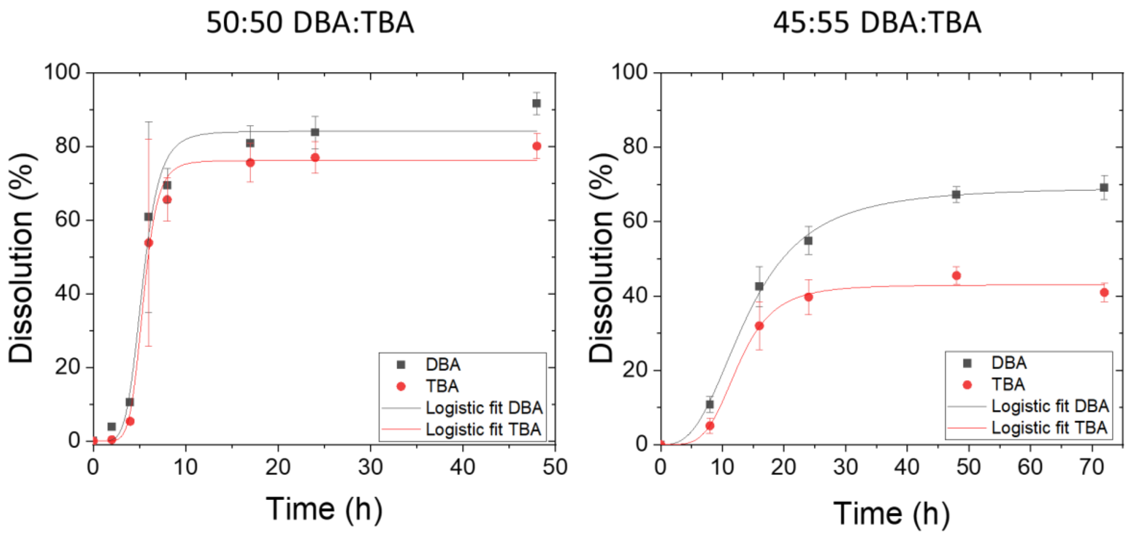

Figure S6.1: Dissolution profile of the (a) 50:50 DBA:TBA system and (b) 45:55 DBA:TBA system with the logistic function fitting.

Function	Logistic			
Equation	$y = A_2 + \frac{A_1 - A_2}{1 + \left(\frac{x}{x_0}\right)^p}$			
Formulation (DBA:TBA)	50:50		45:55	
Plot	DBA	TBA	DBA	TBA
A_1	0±0	0±0	0±0	0±0
A_2	84.15±3.25	76.20±2.00	69.31±1.33	43.01±1.32
x_0	5.31±0.26	5.42±0.17	14.06±0.44	12.60±0.68
p	5.71±1.27	7.08±1.26	2.80±0.22	4.33±0.69
r^2	0.98	0.99	0.99	0.99

Table S6.1: Fitting parameters for logistic function by OriginPro 2024.

S7. <u>Dissolution profiles of the fabrics showing DBA and hydrolyzed di-block separately</u>

The first mesophase transition, from solid fabric to micelles, is independent of the presence of PLE in the system, as the enzyme does not penetrate the fibers to reach the ester bonds. Instead, it hydrolyzes them only when the polymers are in their "monomeric" form. Therefore, in analyzing this transition, we summed the concentrations of DBA and the hydrolyzed di-block to focus solely on fabric dissolution. However, for completeness, we also present the data where DBA and the hydrolyzed di-block are not summed (Figure S7.1).

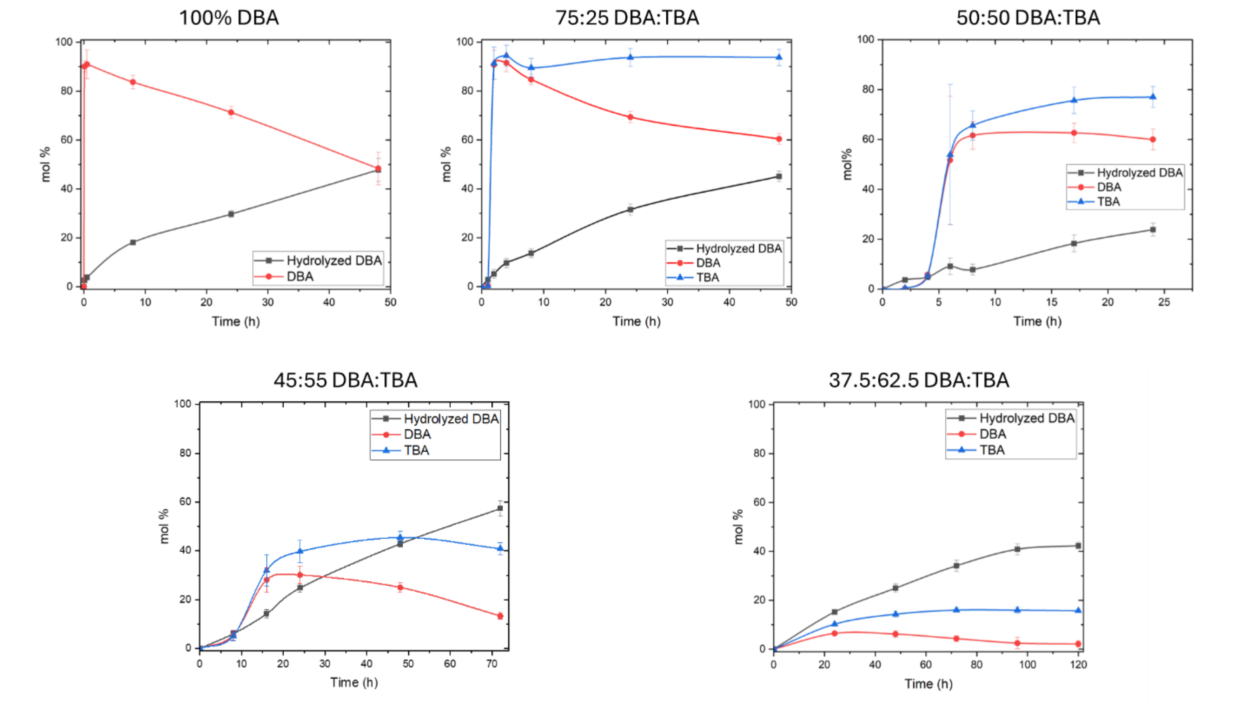

Figure S7.1: Dissolution profiles of the different fabrics showing the concentration of DBA and hydrolyzed di-block separately.

S8. <u>DLS of PLE in PBS solution</u>

In order to understand the background of the DLS measurements caused by the PLE, a blank solution of 3µM of PLE in PBS was measured in the DLS, and the spectrum is shown in Figure S8.1.

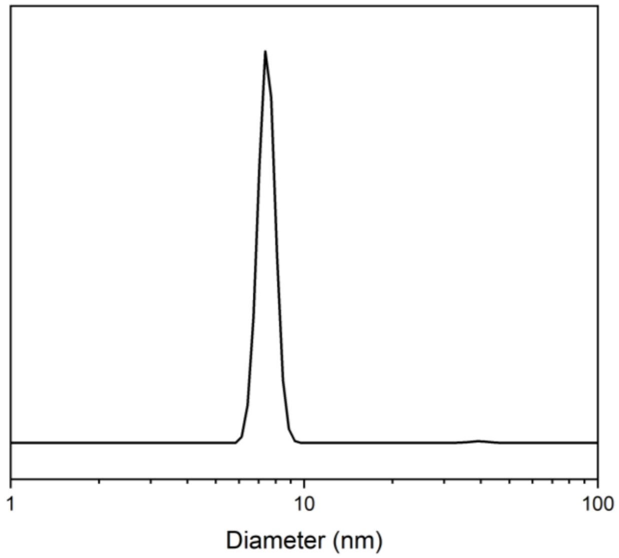

Figure S8.1: DLS measurement of PLE in PBS solution.

S9. <u>Micelles analysis by Transmission Electron Microscopy (TEM)</u>

According to the fibers dissolution analysis procedure, after micelles formation, detected by DLS, the sample was filtered through 0.22µm nylon filter, and 10 µL were loaded on carbon film 200 mesh Cu (TED PELLA, INC). Figure S9.1 shows TEM images for micelles formed for the different ratios: 100% DBA, 75:25 DBA:TBA, 50:50 DBA:TBA, 45:55 DBA:TBA, 37.5:62.5 DBA:TBA.

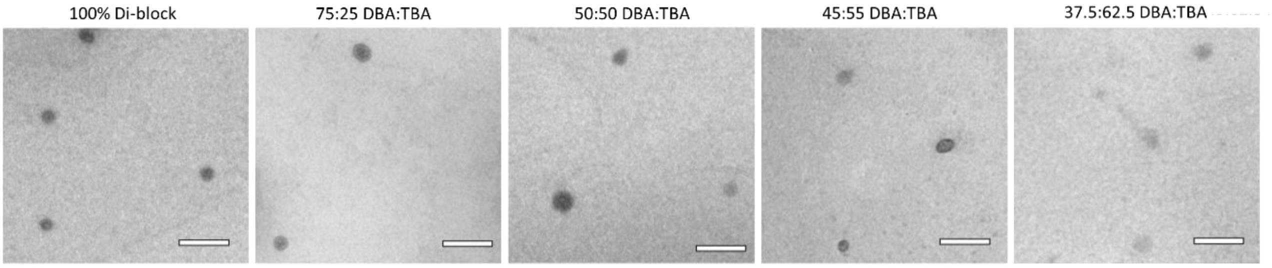

Figure S9.1: TEM images of the micelles formed using different ratios of DBA and TBA, the scale bar represents 100nm.

S10. Fabrics with more than 75% TBA dissolution measurements

In the cases of fabrics with 25:75 DBA:TBA and only TBA that were soaked separately in PBS with 3µM PLE solution, no polymers were obtained in the solution after 2 weeks (Figure S10.1).

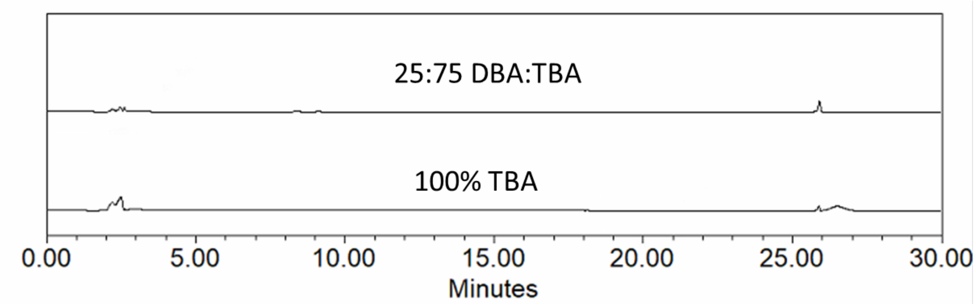

Figure S10.1: HPLC graphs of the solution of the 100% TBA and 25:75 DBA to TBA ratio fabrics after 2 weeks.

S11. Critical Micelle Concentration (CMC) Measurements

Instrument: TECAN Infinite M200Pro device

Excitation wavelength: 550nm

Emission intensity scan: 580-800nm

Diluent solution preparation:

Into 100ml Phosphate-buffered saline (pH 7.4), 45µL of Nile red stock solution (0.88mg/ml in ethanol) were added and mixed to give a final concentration of 1.25µM.

CMC measurement: 500µM solutions of the different ratios were prepared in diluent. The solutions were vortexed and sonicated (25 minutes) until getting clear. This solutions were repetitively diluted by a factor of 1.5 with the diluent. 150µL of each solution were loaded onto a 96 wells plate and a fluorescence emission intensity was performed for each well. The maximum emission intensity at 630nm was plotted vs. the concentration. All measurements were repeated thrice for each formulation.

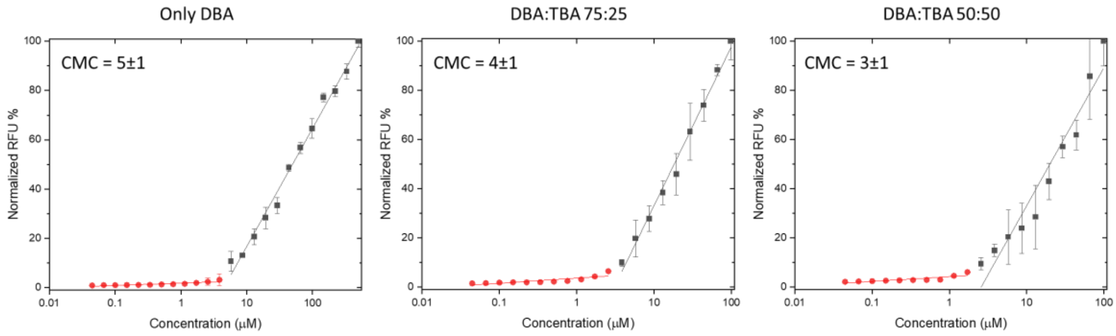

Figure S11.1: CMC measurements of the different formulations.

S12. Fabrics dissolution control measurements without PLE addition

To understand whether the first transition, from fabric to micelles, is affected by the enzyme, control experiments were performed with and without the addition of the enzyme. The degradation was monitored by HPLC at 37°C. In this study, no enzymatic-induced hydrolysis of the polymers occurred, and it was found that the dissolution rate was hardly changed by the presence of the enzyme (Figure S12.1), concluding that the fabric dissolution is not enzymatically induced.

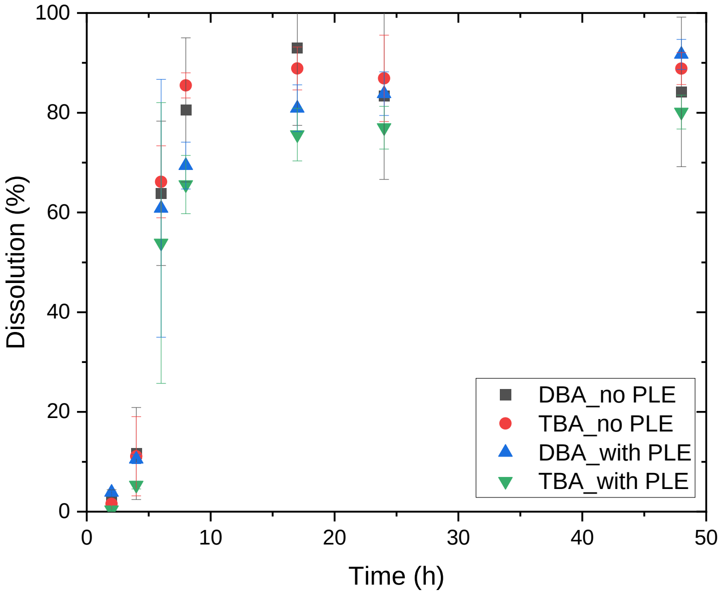

Figure S12.1: HPLC kinetics of the dissolution of the 50:50 DBA to TBA ratio fabric with and without the presence of PLE.

S13. **Hydrogel characterization**

Hydrogel composition analysis by HPLC

To determine the hydrogel's composition for the 50:50 DBA:TBA formulation, the obtained hydrogel was washed with PBS solution three times and then dissolved in acetonitrile. The composition of a clear solution of the dissolved hydrogel was tested by HPLC. Figure S13.1 shows the HPLC chromatogram of the dissolved TBA-based hydrogel implying the presence of 75% TBA, 8% DBA, 2% partly hydrolyzed amphiphiles, 2% fully hydrolyzed tri-block, and 13% fully hydrolyzed di-block.

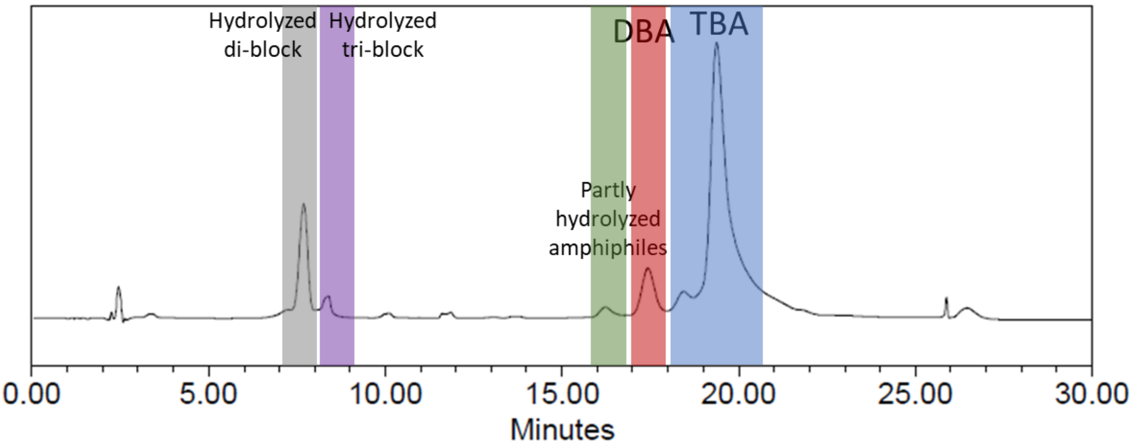

Figure S13.1: HPLC results for the dissolved hydrogel obtained from the 50:50 DBA:TBA system.

Hydrogel Scanning Electron Microscopy (SEM) images

According to the enzymatic micelles degradation procedure, after the hydrogel was observed the solution on top of the hydrogel was taken out, and the sample was lyophilized and observed using SEM.

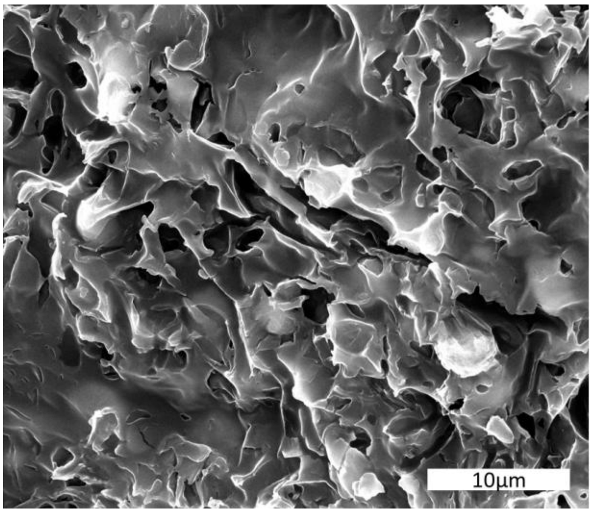

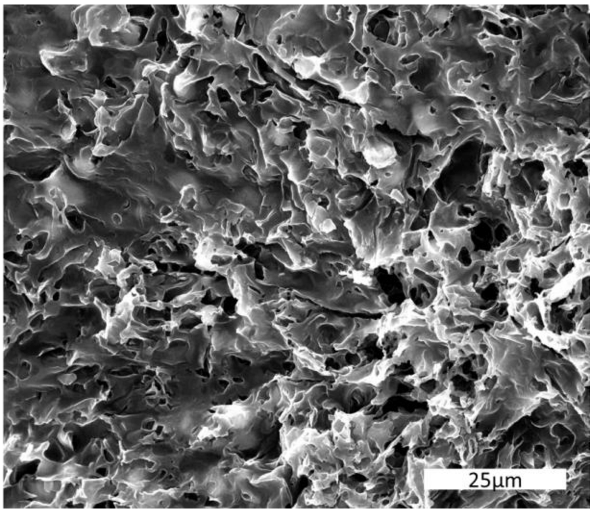

Figure S13.2: SEM images of the lyophilized hydrogel.

S14. <u>Nile red solubility in the last mesophase</u>

When monitoring the encapsulated Nile red in the last mesophase transition from hydrogel to dissolved polymers, we tested the solubility of Nile red in two different solutions. The first is the solution that was inserted into the vial with the hydrogel, PLE (20µM) and BSA (50 mg/mL) solution in PBS. Nile red is insoluble in this solution as can be observed in Figure S14.1.a. The second solution contains the same components, PLE and BSA, with the addition of hydrolyzed tri-block in PBS. The presence of the hydrolyzed tri-block in the solution increases the Nile red solubility as presented in Figure S14.1.b.

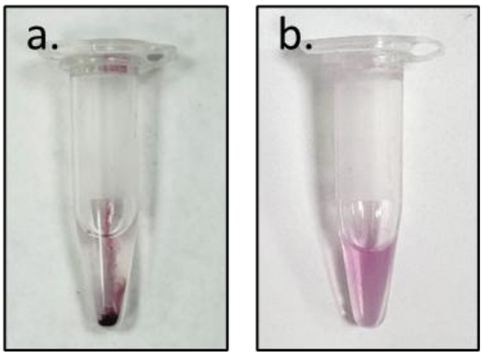

Figure S14.1: Dissolution of Nile red in (a) PLE and BSA solution in PBS and (b) in a hydrolyzed tri-block, PLE, and BSA solution in PBS at 37°C.

References

(1) Segal, M.; Avinery, R.; Buzhor, M.; Shaharabani, R.; Harnoy, A. J.; Tirosh, E.; Beck, R.; Amir, R. J. Molecular Precision and Enzymatic Degradation: From Readily to Undegradable Polymeric Micelles by Minor Structural Changes. *J. Am. Chem. Soc.* **2017**, *139* (2), 803–810. DOI: 10.1021/jacs.6b10624.

(2) Edelstein-Pardo, N.; Molco, M.; Rathee, P.; Koren, G.; Tevet, S.; Sharabani, S. Z.; Beck, R.; Amir, R. J.; Sitt, A. Anisotropic Microparticles through Periodic Autofragmentation of Amphiphilic Triblock Copolymer Microfibers. *Chem. Mater.* **2022**, *34* (14), 6367–6377. DOI: 10.1021/acs.chemmater.2c00859.

(3) DeLean, A.; Munson, P. J.; Rodbard, D. Simultaneous Analysis of Families of Sigmoidal Curves: Application to Bioassay, Radioligand Assay, and Physiological Dose-Response Curves. *Am. J. Physiol. Metab.* **1978**, *235* (2), E97. DOI: 10.1152/ajpendo.1978.235.2.E97.

(4) Ritz, C. Toward a Unified Approach to Dose–Response Modeling in Ecotoxicology. *Environ. Toxicol. Chem.* **2010**, *29* (1), 220–229. DOI: 10.1002/etc.7.

(5) Ritz, C.; Baty, F.; Streibig, J. C.; Gerhard, D. Dose-Response Analysis Using R. *PLoS One* **2015**, *10* (12), e0146021. DOI: 10.1371/journal.pone.0146021.

(6) O'Connell, M. A.; Belanger, B. A.; Haaland, P. D. Calibration and Assay Development Using the Four-Parameter Logistic Model. *Chemom. Intell. Lab. Syst.* **1993**, *20* (2), 97–114. DOI: 10.1016/0169-7439(93)80008-6.
